# How to integrate individual patient values and preferences in clinical practice guidelines? A research protocol

**DOI:** 10.1186/1748-5908-5-10

**Published:** 2010-02-02

**Authors:** Trudy van der Weijden, France Légaré, Antoine Boivin, Jako S Burgers, Haske van Veenendaal, Anne M Stiggelbout, Marjan Faber, Glyn Elwyn

**Affiliations:** 1 Department of General Practice, Maastricht University, School of Public Health and Primary Care (CAPHRI), Maastricht, the Netherlands; 2 Department of Family Medicine, Université Laval, Québec, Canada; 3 Department IQ Healthcare, Radboud University Nijmegen Medical Centre, Nijmegen, the Netherlands; 4 Department of Clinical Practice Guidelines and Indicator Development, Dutch Institute for Healthcare Improvement (CBO), Utrecht, the Netherlands; 5 Department of Medical Decision Making, Leiden University Medical Centre, Leiden, the Netherlands; 6 Department of Primary Care Public Health, Cardiff University, Cardiff, UK

## Abstract

**Background:**

Clinical practice guidelines are largely conceived as tools that will inform health professionals' decisions rather than foster patient involvement in decision making. The time now seems right to adapt clinical practice guidelines in such a way that both the professional's perspective as care provider and the patients' preferences and characteristics are being weighed equally in the decision-making process. We hypothesise that clinical practice guidelines can be adapted to facilitate the integration of individual patients' preferences in clinical decision making. This research protocol asks two questions: How should clinical practice guidelines be adapted to elicit patient preferences and to support shared decision making? What type of clinical decisions are perceived as most requiring consideration of individual patients' preferences rather than promoting a single best choice?

**Methods:**

Stakeholders' opinions and ideas will be explored through an 18-month qualitative study. Data will be collected from in-depth individual interviews. A purposive sample of 20 to 25 key-informants will be selected among three groups of stakeholders: health professionals using guidelines (*e.g*., physicians, nurses); experts at the macro- and meso-level, including guideline and decision aids developers, policy makers, and researchers; and patient representatives. Ideas and recommendations expressed by stakeholders will be prioritized by nominal group technique in expert meetings.

**Discussion:**

One-for-all guidelines do not account for differences in patients' characteristics and for their preferences for medical interventions and health outcomes, suggesting a need for flexible guidelines that facilitate patient involvement in clinical decision making. The question is how this can be achieved. This study is not about patient participation in guideline development, a closely related and important issue that does not however substitute for, or guarantee individual patient involvement in clinical decisions. The study results will provide the needed background for recommendations about potential effective and feasible strategies to ensure greater responsiveness of clinical practice guidelines to individual patient's preferences in clinical decision-making.

## Introduction

Despite the fact that explanation of pros and cons of all available diagnostic and treatment options including doing nothing (the fundamentals of shared decision making) is legally prescribed in some countries [1], it has not been broadly adopted yet in clinical practice [2,3]. Improvement in this area has been observed [4], but active patient involvement in decision making is clearly not easily established.

Clinical practice guidelines (CPG) are systematically developed statements to assist practitioners and patient decisions about appropriate healthcare for specific circumstances [5]. Clinical practice guidelines are an established tool for quality improvement in clinical practice. Although it was suggested years ago to include individual patient values and preferences in clinical practice guidelines [6-9], this is not structurally adopted in current guidelines [10-14]. Clinical practice guidelines are still largely conceived as tools that will inform health professionals' decisions rather than foster patient involvement in decision making [15]. We hypothesise that guidelines can be adapted to facilitate the integration of individual patients' preferences in clinical decision making.

Guidelines are systematically developed in (multidisciplinary) consensus groups, with grading, interpretation, and translation of evidence into recommendations [16]. Guidelines should be looked upon as instruments to guide professionals and should not lead to cookbook medicine [17]. Benchmarks for adherence to recommendations vary, and we know that a certain level of inter-professional variation in adherence to clinical practice guidelines is justified by differences in case mix. Professionals are responsible for adjusting their clinical decisions to each unique individual patient. Relevant arguments (*e.g*. co-morbidity, gender, genetic susceptibility, allergies, the private situation), can justify non-adherence to guidelines. Guideline recommendations are more easily aligned with what is good for a specific population than for a given individual.

Next to patients' individual characteristics, their preferences for interventions should be taken into account in guideline use. Firstly, there is a purely patient-driven argument for incorporating patients' preferences in guideline use related to ethical considerations about patient autonomy. Patients increasingly want to be informed by their doctors [18] and be active in clinical decision-making [19,20], although the wish to actually participate in treatment decision is context-dependent [21].

Second, sound medical evidence is only available for a subset of the recommendations in CPG. In 'preference-sensitive' or 'grey zone' decisions, the lack of evidence results in high levels of uncertainty about the best course of action [22].

Third, even when recommendations are built on rigorous evidence, individuals often vary widely in their preferences, despite the certainty of effect from population-based research. This is the case for the treatment of atrial fibrillation, a well-documented risk factor for stroke. There is a trade-off between the well-known protective effect of oral anticoagulation (warfarin) on stroke, and the increased risk of bleeding. This makes decision making complex, even more so because patients and physicians differ in their preferences for management of atrial fibrillation [23-25].

Fourth, even when high-quality evidence is available more than one effective treatment options may co-exist, with comparable effectiveness of the various options from a medical point of view. This is referred to as 'equipoise' [26]. These options may be equal in the sense that scientific evidence points to a balance between harms and benefits within or between options. For example, aspirin is an alternative therapy for prevention of stroke in atrial fibrillation, less effective compared to warfarin, but also with much lower risk of bleeding.

Fifth, there is evidence that patient preferences and motivation for treatment positively affect treatment outcomes in randomized controlled trials (RCTs) in musculoskeletal medicine [27].

In conclusion, one-for-all guidelines that are designed for a specific population do not account for differences between patients' characteristics and preferences, suggesting a need for flexible guidelines that enable and facilitate patient involvement in medical decision making.

In this study, patient preference is defined as the appraisal of an individual who is informed and knowledgeable about the probabilities and severity of the effects and risks of interventions, and about process and outcome aspects of healthcare. For example, a choice may be made between a surgical or a pharmaceutical approach in treating a disease, or between taking up or not taking up a preventive measure for which there is considerable uncertainty in effect, or between consenting or not consenting to a more intensified treatment in the case of chronic disease.

Patients will not seek involvement in decisions for which it is evident what needs to be done (*e.g*. in urgent situations such as accidental hip fracture). Many decisions are preference-sensitive, or one could even say, all decisions are preference-sensitive, because a patient can always opt for doing nothing. Nevertheless, some recommendations within clinical practice guidelines are more preference-sensitive than others, such as decisions with lifelong implications on chronic disease management, or interventions carrying an important risk or with uncertain benefit [28,29]. The process of developing clinical practice guidelines is expensive, and can be in the order of several €100,000 [9]. The complexity and costs of this process may increase further if we implement strategies for involving patients in decision making, such as the development of patient decision aids as part of the guideline. Therefore, a sober attitude towards full-blown integration of shared decision-making strategies into guidelines seems justified. Clinical practice guidelines should recommend elicitation of patient values at specific decision points [30,31]. Various crude criteria have been described to select preference-sensitive decisions on diagnostic or therapeutic interventions within a CPG [8,11,13,32-34], e.g.: unclear or conflicting evidence; the intervention involves risks or side effects; the intervention affects quality of life rather than length of life; a published patient decision aid (from another country) is available; financial considerations for the patient (out-of-pocket costs); or the recommendation is rated as highly important by patients but not by doctors. It is not clear, however, what exactly are these specific preference-sensitive decision points, what criteria should have priority to be used to label a decision as preference-sensitive, and how this should be done in guideline documents.

This study protocol is not about the active participation of patients in the process of CPG development (collective perspective of 'the patient'), but on how CPG can be improved to stimulate the consideration of individual patient values and preferences during the physician-patient contact (individual preference-flexible approach). Currently, much attention is devoted to innovative methods to engage patient and public representatives in the process of guideline development, which is seen as important by patient groups and guideline developers [35]. Patients' collective norms and values are considered in the interpretation of medical evidence and its translation into recommendations [36,37]. Patient participation in guideline development can support collective decisions about healthcare organization and delivery. This may lead to important adjustments to guideline documents, for example in broadening the patient outcomes that are considered in the guideline [38,39].

However, patient participation in CPG development, which is an important innovation in itself, is not substitute for involvement of patients or consumers in individual clinical decisions. Indeed, patient representatives cannot be expected to provide input on what 'the patient' with a particular disease prefers and what 'the patient' experiences.

Although they represent an important adaptation of CPG development process, relying solely on collective involvement approaches will probably not be sufficient to optimise guideline responsiveness to individual patient's preferences [8,40,41].

The aim of this paper is to describe a protocol for an explorative study on strategies for the integration of individual patient's preferences in decision making based on clinical practice guidelines. Our research questions are:

1. How should clinical practice guidelines be adapted to elicit individual patients' preferences and to support patients' and health professionals' shared decision making? For example: How should clinical practice guidelines and patient decision support technology be linked, and what are barriers and facilitators for doing so?

2. What types of clinical decisions are perceived by stakeholders as most requiring consideration of preferences of individual patients rather than promoting a single best choice?

To limit the magnitude of these research questions and to facilitate data collection the research questions are applied to two concrete examples of preference-sensitive decisions: the decision to prescribe or not to prescribe anti-depressive drugs on top of cognitive behavioural therapy for a patient diagnosed with a major depression; and the decision between ablation or lumpectomy for a women diagnosed with breast cancer.

## Methods

### Study design

Empirical studies on this issue are scarce in the indexed literature [41]. Therefore, we chose to explore stakeholders' opinions and ideas in an 18-month qualitative study, beginning in mid-2009: Data will first be collected from in-depth individual interviews. Ideas and recommendations expressed by stakeholders will be prioritized by nominal group technique in expert meetings.

The Maastricht Medical Research Ethics Committee approved that this study does not fall under the medical ethics law.

### Theoretical background

The theoretical background of this study is found in shared decision making and implementation science. The most generally accepted conceptualization of shared decision making is that of Charles *et al*., who identified the key features of shared decision making as involvement of both the patient and doctor, a sharing of information by both parties, both parties taking steps to build a consensus about the preferred treatment, and reaching an agreement about which treatment to implement [42,43].

Grol has described a general model for implementation of guidelines or innovations in which a systematic approach as well as good preparation and planning are central issues [44]. The implementation strategies can be focused at the individual care provider (knowledge, attitude, motivation to change, personal characteristics), at the social setting (other care providers and patients), or at the organisational and financial system. Not surprisingly, the guideline can have a major impact on the success of implementation [45]. In this project, we focus on the level of guideline use in clinical practice to enhance implementation of shared decision making by improving clinical practice guidelines.

### Population in-depth interviews

We will recruit a purposive sampling of 20 to 25 key-informants. We aim for a heterogeneous sample of participants with different perspectives and ideas on how to incorporate individual patient's preferences in guidelines. We will identify contextual factors that influence the stakeholders' perception of what is a preference-sensitive decision. We will select interview participants from three stakeholder groups: professional users of CPG (physicians, nurses); experts at the macro- and meso-level (policy makers, CPG development organisations, decision aid developers, researchers); and patient representatives.

Professionals/guideline users (physicians and nurses): we will include four to six opinion leaders who have a special interest in one of the selected clinical areas. Policy makers/guideline/decision aid developers/researchers: we will include four to six members of guideline development institutions to be recruited in or via the steering group of the Guidelines International Network Patient and Public Involvement Working Group (G-I-N PUBLIC), and four to six members of the International Patient Decision Aids Standard collaboration (IPDAS) (patient decision aid developers, researchers). Finally, we will recruit at least six patients who closely collaborate with relevant national patient and quality improvement institutions, such as the Dutch patient and consumer federation (NPCF) and the Dutch Institute for Healthcare Improvement (CBO), as well as patients representing people with the chronic conditions that will be chosen as illustrative examples (depression and breast cancer). Non-Dutch patient representatives from G-I-N PUBLIC or the Cochrane Consumer Network will be approached as well.

### Data collection interviews

The semi-structured interview scheme will start with open questions and is adapted to each stakeholder group. The face-to-face or phone interviews will be open and will be characterized by a personal approach, meaning that the interviewer has some knowledge of the background and work of the person to be interviewed, ensures that the objective and procedure of the study are clear, and stimulates the participant to express his or her opinion by explaining that there are no good or wrong answers and that each opinion or idea will be included in the analysis. After the introduction, the interviewers will follow the interview scheme, based on a list of themes and examples to ensure that all relevant items are brought up during the discussion. All interviews will be audio-taped, and transcribed verbatim. Experienced and independent senior researchers will carry out the interviews.

Member-checking will be done to validate our analysis by sending interview participants a summary of the main findings, extracted from a single interview. The participant will be contacted by phone or email for his or her reaction, to prevent any misunderstanding in the transcribing or interpretation.

### The interview scheme

Based on the literature (see Appendix 1) and experiences of the project group members, a semi-structured interview scheme will be developed for practising professionals, policy makers/guideline developers/researchers, and for patients. The interview scheme will be used in a formative way and adapted during the data collection on the basis of the interviews' findings.

The interviewees do not have to prepare for the interview, but a package, either about depression or breast cancer, will be sent to them two weeks in advance of the interview. The package contains a one-page summary of the decision at stake, including a fact sheet describing benefits and risks for each option, as well as a copy of the current national clinical practice guideline on the specific clinical subject. During the interview, other information may be shared with the interviewee, depending on the content of the interview, such as: a summary of empirical evidence on the preferences of fully informed patients about the depression or breast cancer decision, or a patient decision aid. In the summary, only the most relevant available empirical literature on patient preferences for the selected topics will be given.

The interview scheme is not a checklist that has to be followed in this order, but functions as a guide for topics to be mentioned whenever it seems most suitable in the flow of the interview. It will cover the following topics:

1. Introduction and informed consent: Explanation of the aim of the interview (standardised text read aloud by the interviewer), asking for informed consent, explaining the anonymous character of data analysis, and permission for audio-taping the interview.

2. Open question on the interviewee's views on the subject.

3. Topics related to research question one (strategies to integrate individual patient preferences in guidelines for these most urgent preference-sensitive decisions): Depending on the course of the interview and prompted by the input of the interviewee, the interviewer reflects on the evolving overview of strategies to integrate individual patient preferences in guidelines currently described in the literature, and stimulates the interviewee to respond on other strategies.

Special attention will be given to strategies to link guidelines and patient decision aids: The following illustrative example might be given: The CBO has extended patient participation in guideline development with producing patient decision aids as co-products for CPG [32]. This may be followed by more specific questions, prompted by the input of the interviewee. If suitable, they may be asked to respond on the overlap in two sets of quality criteria; the Appraisal of Guideline Research and Evaluation collaboration (AGREE) [46] and the IPDAS [47].

4. Topics related to research question two (labelling the most urgent preference-sensitive decisions during the review of the evidence as part of the guideline development): Respondents will be asked to think of decisions that they think individual patients should preferably be invited and stimulated to be involved in, and share the decision with the professional, and to reflect on the reasons that support their view.

An example of a specific question that may emerge is on the potential role of the Grades of Recommendation Assessment, Development and Evaluation (GRADE) system [48]. The GRADE system for rating the quality of evidence and strength of recommendations describes four factors that affect the strength of a recommendation: 1. the quality of evidence (homogeneous meta-analysis versus case studies); 2. uncertainty about the balance between desirable and undesirable effects (low versus high levels of toxicity, inconvenience, costs); 3. uncertainty or variability in values and preferences (young patients value the trade-off between life-prolonging effects and treatment toxicity of chemotherapy differently compared to old patients); 4. uncertainty about whether the intervention represents a wise use of resources (low versus high costs, favourable versus unfavourable budget impact analysis). Strong recommendations mean that most informed patients would choose the recommended management, and that clinicians can structure their interactions with patients accordingly. Weak recommendations mean that patients' choices will vary according to their values and preferences, and clinicians must ensure that patients' care is in keeping with their values and preferences. Are the GRADE criteria at all useful in labelling urgent preference-sensitive decisions? Should all weak recommendations be signalled as preference-sensitive decisions? Or should there be a ranking of more and less urgent preference-sensitive decisions based on the third GRADE factor?

### Data analysis

The interviews will be analysed by directive content analysis [49]. Data will be collected and analysed concurrently, allowing both expected and emergent themes and ideas to be incorporated and explored in subsequent interviews. The data will be divided into simpler text units for coding that will be entered into a database (Atlas or Nvivo). Units of text referring to similar codes will be grouped and categorized systematically by one central coder, who is coding all the interviews. For the most informative interview--in the opinion of the interviewer--of each subset of interviews, a full open coding of the transcript will be independently executed by the central coder and the interviewer. Differences in coding will be resolved by consensus discussion face-to-face or by phone. The central coder will then analyse the other interviews, of the subset of interviews done by the one interviewer, and the interviewer will check the coding. Major differences in interpretation in codes will be solved by email and telephone contact.

### Validation and prioritisation of the final recommendations

Two expert meetings will be organised to validate findings and prioritize recommendations: one among experts in the Netherlands, and one executed at an international conference. Expert-meetings will also aim to formulate recommendations for guideline developers, and to set a research agenda. The data from the individual interviews will be triangulated with experts' opinions. We will apply the four phases of the nominal group technique for this expert meeting:

1. In the first 'generating ideas' phase, the moderator explains the procedure and asks participants to prioritize the proposed list of recommendations and to write down the main research questions that follow to evaluate the effectiveness of incorporating patient preferences in clinical practice guidelines.

2. In the second 'recording' phase, each group's members will be engaged in a round-robin feedback session to concisely record each idea. Priorities and research questions will be noted and numbered on flip charts.

3. In the next 'evaluation' phase, each recorded idea will be discussed to obtain clarification and evaluation. Group members will participate in the process of clarification, and of weighing the pros and cons of the proposed ideas.

4. The purpose of the last phase is to aggregate the judgments of individual members to determine the relative importance of the ideas. In this phase, the individual experts vote privately on the priority of ideas, and a group decision will be made based on these ratings.

The international expert meeting will be held in August 2010 at the Guidelines International Network (G-I-N) conference. We will purposively sample well-known opinion leaders and experts, with the aim to have eight to ten experts who volunteer to participate. The (para-)medical professionals, as principal guideline users, should be well-represented. The sessions will be chaired by an experienced moderator, assisted by one of the project members using flip charts. We will develop a scenario in advance to ensure that all phases of Nominal Group Technique will be completed.

The participants will be given the results of the interviews two weeks before by email or post. We will provide a draft version of the report/analysis with extended quotes supporting the analysis in footnote or tables. The results will also be summarized in a list of 'do's and don'ts'.

### Time schedule

Phase one (months one to four): Exploratory conference workshops and development of the interview scheme. The literature and experiences available to the project team are critically reviewed to generate input for the interview scheme.

Phase two (months five to fourteen): Semi-structured interviews with stakeholders' groups and concurrent data analysis.

Phase three (months fifteen to eighteen): Validation and prioritisation of findings at expert meetings.

## Discussion

In this study, we seek to find answers to questions about how clinical practice guidelines can be developed to guarantee more sensitivity to individual patient's preferences during decision making in the consultation room. This study may lead to recommendations for potential effective strategies that can be used by guideline development institutions and made available to patient's groups. Our goal is to generate input for the development of one or two promising, feasible, and efficient strategies for incorporation of individual patient preferences into clinical practice guidelines. In the future, we aim to design a follow-up study in which these different types of guidelines (more and less sensitive for individual patient preferences) are evaluated in an experimental design, with the process of decision making being used as the primary outcome.

### Strengths and limitations

The strength of this study is the use of a combination of different qualitative methods (interviews, literature search, and nominal group technique). These will generate input for the development of effective and feasible strategies for making guidelines sensitive to individual patients' preferences. Another strength is the international character of the study, ensuring that many different viewpoints will be considered. In addition, the composition of the multidisciplinary project group--representing disciplines such as general practice, epidemiology, health technology assessment, health science, and implementation science--will minimise bias towards a specific theoretical perspective. There is strong collaboration with our colleagues from Canada who are executing a realist review of strategies for patient participation in guideline development and implementation [36].

Our selection of interviewees is based on information from the literature, the personal network of the project group members, and pragmatic reasons such as attendance at an international conference. We estimate that with the planned number of interviewees data saturation will be reached.

### Other considerations

In the definition of evidence-based medicine (EBM), thoughtful identification and compassionate use of individual patients' preferences in making clinical decisions is promoted. EBM is the conscientious, explicit, and judicious use of current best evidence in making decisions about the care of individual patients [50]. Nevertheless, EBM guidelines are often viewed as conflicting with patient-centred medicine and with taking into account individual choice and preference [51]. Clinical practice guidelines are typically derived from population-based studies and perceived as limiting patient's choice by advocating only one appropriate course of action. The constructionist critique of EBM is about 'evidence' being more an artefact rather than 'reality'. It is argued that research interests, activity driven by historical contingencies, and powerful commercial interests (mostly new pharmaceutical products) steer EBM agenda's instead of focussing on investigating the complex processes of healthcare delivery that are of greatest importance to patients [52]. Therefore, the underlying assumption in this proposal, to facilitate patient involvement or even shared decision making during the consultation by means of adapting guidelines, might be provocative for those who regard EBM and clinical practice guidelines as being in conflict with patient-centred care. Nevertheless, we feel that this is the right time to take up the challenge and to see how such established tools like guidelines can be adapted in such a way that evidence-based guideline recommendations, professional expertise, the context of the individual patient and practice situation, and patients' preferences and autonomy are being equally weighed in the decision-making process.

This study proposal is closely related to the innovations in patient participation in guideline development. Bastian was one of the first to attract attention to patient participation in guideline development [53]. Boivin illustrated that the exact purpose of involving patients in this process is not straightforward. He identified four discourses on the goal and meaning of considering patient preferences in clinical practice guidelines; the governance discourse, the informed decision discourse, the professional care discourse, and the consumer advocacy discourse [54].

Although we have described a distinction between 'collective' and 'individual' approaches to involvement, we recognise that it is not easy to exactly define where collective patient participation ends and strategies for incorporation of individual patient preferences in guidelines begin. Not all forms of patient involvement in guideline development assume the construction of a single patient. One could argue, for example, that patient representatives in guideline development groups could become advocates for more flexible approaches to guideline use and incorporation of decision aids. Hence, collective-level involvement could potentially support the development of 'preference-flexible guidelines'.

Looking at the AGREE and IPDAS criteria, and how these two sets of criteria overlap, gives rise to the question of how IPDAS can be used as criteria for best practices regarding how to communicate evidence to the professionals through guidelines.

A threat that may bring clinical practice guidelines and shared decision making in conflict is the tendency of policy makers and healthcare insurers to introduce incentives for doctors to reach certain practice targets, especially applied at chronic disease management such as diabetes care, without accounting for differences in case mix, co-morbidity, and patient preferences [55]. The aim is to try to improve quality of care, which in itself is a laudable aim, but one which potentially conflicts with patients' rights to be involved in their care and to make choices which may or may not be aligned with what is set down as the standard of care [56].

## Appendix 1. Suggestions in the literature to make CPG more sensitive to individual patient's preferences

Researchers from the field of health technology assessment and decision analysts propose to include decision analytical methods in the CPG, integrating formal utility assessment in the CPG by instructing patients to assign weights or utilities to options [8,55,57]. It is doubted whether measuring utilities should be the way forward [58], and it is not clear how this could be actually done in practice.

Other suggestions are about ways to reveal equipoise in the CPG, *e.g*., by alerting readers to the particular needs of patients [10,11,59], by presenting two equally valid scenarios [60], or presenting a second best scenario as an alternative to the key recommendation [61]. Equipoise can also be revealed by displaying preference and value-related evidence, and including empirical data on patients' actual decisions, whether supported or not supported by patient decision aids [10,13].

Some of the suggestions are about providing recommendations on the level of the decision-making process and concurrent development of patient decision aids by the CPG development group [62]. Patient decision aids are increasingly made available, and there is growing consensus on how decision aids should be constructed [45,63]. Examples of such recommendations are the timely prescription of 'information prescriptions' or referrals to a 'preference laboratory' (places where patients can view decision aids and answer questions about their values preferences) [64], or the recommendation to schedule an extra consultation to help patients to prepare for shared decision making [55]. Some suggestions have been made, ranging from a generic tool for development of patient decision aids based on CPG, preferably developed concurrently by the CPG development group [43], to the integration of risk communication tools as part of CPG [11], and the potential acceleration of this process by the improved access to global information via the internet and worldwide web [65].

## Competing interests

The authors declare that they have no competing interests.

## Authors' contributions

All authors have participated in the design of the study and read and approved the final manuscript. TvdW is applicant and GE is co-applicant of the study grant.

## References

[B1] Weijden T Van der van Veenendaal H Timmermans DRM Shared decision making in the Netherlands Zeitschrift fur Arztliche Fortbildung und Qualitatssicherung 2007 101 241 6 17601179 10.1016/j.zgesun.2007.02.022

[B2] Elwyn G Légaré F Edwards A Weijden T van der May C Arduous implementation: Does the Normalisation Process Model explain why it's so difficult to embed decision support technologies for patients in routine clinical practice Implementation Science 2008 3 57 10.1186/1748-5908-3-57 19117509 PMC2631595

[B3] Légaré F Ratté S Gravel K Graham ID Barriers and facilitators to implementing shared decision-making in clinical practice: update of a systematic review of health professionals' perceptions Pat Educ Couns 2008 73 526 35 10.1016/j.pec.2008.07.018 18752915

[B4] Brink-Muinen A Van den van Dulmen SM de Haes HCJM Visser A Schellevis FG Bensing JM Has patients' involvement in the decision-making process changed over time? Health Expect 2006 9 333 42 10.1111/j.1369-7625.2006.00413.x 17083560 PMC5060368

[B5] Field M Lohr K Clinical practice guidelines: Directions for a new agency 1990 National Academic Press, Washington 25144032

[B6] Eddy DL Rationing by patient choice JAMA 1991 265 105 8 10.1001/jama.265.1.105 1984107

[B7] Gilmore A Clinical practice guidelines: weapons for patients, or shields for MDs? Can Med Ass J 1993 148 429 31 PMC1490488 8439916

[B8] Nease RF Owens DK A method for estimating the cost-effectiveness of incorporating patient preferences into practice guidelines Med Dec Making 1994 14 382 92 10.1177/0272989X9401400409 7808213

[B9] Gandjour A Westenhofer J With A Fuchs C Lauterbach KW Development process of an evidence-based guideline for the treatment of obesity Int J Qual Healthcare 2001 13 325 32 10.1093/intqhc/13.4.325 11560352

[B10] Schünemann HJ Fretheim A Oxman AD Improving the use of research evidence in guideline development: 10. Integrating values and consumer involvement Health Res Policy Syst 2006 4 22 10.1186/1478-4505-4-22 17147811 PMC1697808

[B11] McCormack JP Loewen P Adding 'value' to clinical practice guidelines Can Fam Physician 2007 53 1326 7 17872848 PMC1949258

[B12] Chong C Chen I Naglie C Krahn M Do clinical practice guidelines incorporate evidence on patient preferences? Med Dec Making 2007 27 E63 4

[B13] Krahn M Naglie G The next step in guideline development: incorporating patient preferences JAMA 2008 300 436 8 10.1001/jama.300.4.436 18647988

[B14] Shaneyfelt T Centor R Reassessment of clinical practice guidelines. Go gently into that good night JAMA 2009 301 868 9 10.1001/jama.2009.225 19244197

[B15] Boivin A Légaré F Gagnon MP Competing norms: Canadian rural family physicians' perception of clinical practice guidelines and shared decision-making J Health Services Res Policy 2008 13 79 84 10.1258/jhsrp.2007.007052 18416912

[B16] Guyatt GH Oxman AD Vist GE Kunz R Falck-Ytter Y Alonso-Coello P Schünemann H GRADE: an emerging consensus on rating quality of evidence and strength of recommendations BMJ 2008 336 924 6 10.1136/bmj.39489.470347.AD 18436948 PMC2335261

[B17] Cabana MD Rand CS Powe NR Wu AW Wilson MH Abboud PA Rubin HR Why don't physicians follow clinical practice guidelines? A framework for improvement JAMA 1999 282 1458 65 10.1001/jama.282.15.1458 10535437

[B18] Coulter A Partnerships with patients: the pros and cons of shared clinical decision making J Health Serv Res Policy 1997 2 112 21 10180362 10.1177/135581969700200209

[B19] Kiesler DJ Auerbach SM Optimal matches of patient preferences for information, decision-making and interpersonal behavior: Evidence, models and interventions Pat Educ Couns 2006 61 319 41 10.1016/j.pec.2005.08.002 16368220

[B20] Say R Murtagh M Thomson R Patients' preference for involvement in medical decision making: A narrative review Pat Educ Couns 2006 60 102 114 10.1016/j.pec.2005.02.003 16442453

[B21] Llewellyn-Thomas HA Measuring patients' preferences for participating in healthcare decisions: avoiding invalid observations Health Expect 2006 4 305 6 10.1111/j.1369-7625.2006.00418.x PMC5060367 17083557

[B22] O'Connor AM Using decision aids to help patients navigate the 'grey zone' of medical decision making CAMJ 2007 176 1597 8 10.1503/cmaj.070490 PMC1867846 17515586

[B23] Protheroe J Fahey T Montgomery AA Peters TJ The impact of patients' preferences on the treatment of atrial fibrillation: observational study of patient based decision analysis BMJ 2000 320 1380 4 10.1136/bmj.320.7246.1380 10818030 PMC27382

[B24] Devereaux PJ Anderson DR Gardner MJ Putnam W Flowerdew GJ Brownell BF Differences between perspectives of physicians and patients on anticoagulation in patients with atrial fibrillation: observational study BMJ 2001 323 1218 22 10.1136/bmj.323.7323.1218 11719412 PMC59994

[B25] Alonso-Coello P Montori VM Sola I Schünemann HJ Devereaux PJ Chareles C Roura M Diaz MG Souto JC Alonso R Oliver S Ruiz R Coll-Vinent B Diez AI Gich I Guyatt G Values and preferences in oral anticoagulation in patients with atrial fibrillation, physicians' and patients' perspectives: protocol for a two-phase study BMC Health Serv Res 2008 8 221 10.1186/1472-6963-8-221 18954427 PMC2613147

[B26] Elwyn G Edwards A Kinnersley P Grol R Shared decision making and the concept of equipoise: the competences of involving patients in healthcare choices Br J Gen Pract 2000 50 892 9 11141876 PMC1313854

[B27] Preference Collaborative Review Group Patients' preferences within randomised trials: systematic review and patient level meta-analysis BMJ 2008 337 a1864 10.1136/bmj.a1864 18977792 PMC2659956

[B28] Boivin A Légaré F Lehoux P Decision technologies as normative instruments: exposing the values within Pat Educ Couns 2008 59 426 30 10.1016/j.pec.2008.07.017 18774669

[B29] Joosten EA Defuentes-Merillas L de Weert GH Sensky T Staak CP van der de Jong CA Systematic review of the effects of shared decision-making on patient satisfaction, treatment adherence and health Status Psychother Psychosom 2008 77 219 26 10.1159/000126073 18418028

[B30] Rogers WA Evidence-based medicine in practice: Limiting or facilitating patient choice? Health Expectations 2002 5 95 103 10.1046/j.1369-6513.2002.00168.x 12031050 PMC5060137

[B31] Pieterse AH Baas-Thijssen MCM Marijnen CAM Stiggelbout AM Clinician and cancer patient views on patient participation in treatment decision-making: a quantitative and qualitative exploration Br J Cancer 2008 99 875 82 10.1038/sj.bjc.6604611 18781148 PMC2538766

[B32] Raats CJ van Veenendaal H Versluijs MM Burgers JS A generic tool for development of decision aids based on clinical practice guidelines Patient Educ Couns 2008 73 413 7 10.1016/j.pec.2008.07.038 18768285

[B33] Owens D Patient preferences and the development of practice guidelines Spine 1998 23 1073 9 10.1097/00007632-199805010-00023 9589550

[B34] Schofield M Patient-provider agreement on guidelines for preparation for breast cancer treatment Behavorial Medicine 1997 23 36 45 10.1080/08964289709596365 9201429

[B35] Verkerk K Van Veenendaal H Severens JL Hendriks EJM Burgers JS Considered judgement in evidence-based guideline development Int J Qual Healthcare 2006 18 365 9 10.1093/intqhc/mzl040 16959800

[B36] Légaré F Boivin A Weijden T van der Packenham C Tapp S Burgers J A knowledge synthesis of patient and public involvement in clinical practice guidelines: study protocol Implem Science 2009 4 30 10.1186/1748-5908-4-30 PMC2698931 19497114

[B37] Boivin A Currie K Fervers F Gracia J James M Knaapen L Marshall C Sakala C Sanger S Thomas V Weijden T van der Grol R Burgers JS on behalf of G-I-N Public Patient and public involvement in clinical guidelines: international experiences and future perspectives Accepted Qual Saf Healthcare10.1136/qshc.2009.034835 20427302

[B38] Kirwan JR Minnock P Adebajo A Bresnihan B Choy E de Wit M Hazes M Richards P Saag K Suarez-Almazor M Wells G Hewlett S Patient perspective: fatigue as a recommended patient centered outcome measure in rheumatoid arthritis J Rheumatol 2007 34 1174 7 17477482

[B39] Hoes JN Jacobs JW Boers M Boumpas D Buttgereit F Caeyers N Choy EH Cutolo M Da Silva JA Esselens G Guillevin L Hafstrom I Kirwan JR Rovensky J Russell A Saag KG Svensson B Westhovens R Zeidler H Bijlsma JW EULAR evidence-based recommendations on the management of systemic glucocorticoid therapy in rheumatic diseases Ann Rheum Dis 2007 66 1560 7 10.1136/ard.2007.072157 17660219 PMC2095301

[B40] Coulter A Whatever happened to shared decision-making Health Expect 2002 5 185 6 10.1046/j.1369-6513.2002.00190.x 12199657 PMC5060156

[B41] Bovenkamp HM van de Trappenburg MJ Reconsidering patient participation in guideline development Healthcare Anal 2009 17 198 216 10.1007/s10728-008-0099-3 PMC2725277 19101804

[B42] Charles C Gafni A Whelan T Decision-making in the physician-patient encounter: revisiting the shared treatment decision-making model Soc Sci Med 1999 49 651 661 10.1016/S0277-9536(99)00145-8 10452420

[B43] Edwards A Elwyn G Shared decision-making in healthcare. Achieving evidence-based patient choice 2009 2 Oxford University Press

[B44] Grol R Wensing M Eccles M Improving patient care The implementation of change in clinical practice 2005 Elsevier Publisher 45

[B45] Grol R Dalhuijsen J Thomas S In't Veld C Rutten G Mokkink H Attributes of clinical guidelines that influence use of guidelines in general practice: observational study BMJ 1998 317 858 61 9748183 10.1136/bmj.317.7162.858 PMC31096

[B46] Burgers JS Grol R Klazinga NS Mäkelä M Zaat J AGREE Collaboration Towards evidence-based clinical practice: an international survey of 18. clinical guideline programs Int J Qual Healthcare 2003 15 31 45 10.1093/intqhc/15.1.31 12630799

[B47] Elwyn G O'Connor A Stacey D Volk R Edwards A Coulter A Thomson R Barratt A Barry M Bernstein S Butow P Clarke A Entwistle V Feldman-Stewart D Holmes-Rovner M Llewellyn-Thomas H Moumjid N Mulley A Ruland C Sepucha K Sykes A Whelan T International Patient Decision Aids Standards (IPDAS) Collaboration. Developing a quality criteria framework for patient decision aids: online international Delphi consensus process BMJ 2006 333 417 10.1136/bmj.38926.629329.AE 16908462 PMC1553508

[B48] Guyatt GH Oxman AD Kunz R Falck-Ytter Y Vist GE Liberati A Schünemann HJ GRADE: going from evidence to recommendations BMJ 2008 336 1049 51 10.1136/bmj.39493.646875.AE 18467413 PMC2376019

[B49] Hsieh HF Shannon SE Three approaches to qualitative content analysis Qual Health Research 2005 15 1277 88 10.1177/1049732305276687 16204405

[B50] Sackett DL Rosenberg WM Gray JA Haynes RB Richardson WS Evidence based medicine: what it is and what it isn't BMJ 1996 312 71 2 8555924 10.1136/bmj.312.7023.71 PMC2349778

[B51] Bensing J Bridging the gap. The separate worlds of evidence-based medicine and patient-centered medicine Pat Educ Couns 2000 39 17 25 10.1016/S0738-3991(99)00087-7 11013544

[B52] Pollock K Concordance in medical consultations. A critical review 2005 Radcliffe Publ. Abingdon UK

[B53] Bastian H Raising the standard: practice guidelines and consumer participation Int J Qual Healthcare 1996 8 485 90 10.1093/intqhc/8.5.485 9117202

[B54] Boivin A Green J Meulen J van der Légaré F Nolte E Why consider patients' preferences? A discourse analysis of clinical practice guideline developers Med Care 2009 47 908 15 10.1097/MLR.0b013e3181a81158 19543120

[B55] Boyd CM Darer J Boult C Fried LP Boult L Wu AW Clinical practice guidelines and quality of care for older patients with multiple comorbid disease: implications of pay for performance JAMA 2005 294 716 24 10.1001/jama.294.6.716 16091574

[B56] Baratt A Evidence based medicine and shared decision making: The challenge of getting both evidence and preferences into healthcare Pat Educ Couns 2008 73 407 12 10.1016/j.pec.2008.07.054 18845414

[B57] Brennan PF Strombom I Improving healthcare by understanding patient preferences: the role of computer technology J Am Med Inform Ass 1998 5 257 62 10.1136/jamia.1998.0050257 PMC61299 9609495

[B58] Elwyn G Edwards A Eccles M Rovner D Decision analysis in patient care Lancet 2001 358 571 4 10.1016/S0140-6736(01)05709-9 11520546

[B59] McInnes E Cullum N Nelson EA Luker K Duff LA The development of a national guideline on the management of leg ulcers J Clin Nursing 2000 9 208 17 10.1046/j.1365-2702.2000.00369.x 11111612

[B60] Oppenheim PI Sotiropoulos G Baraff LJ Incorporating patient preferences into practice guidelines: management of children with fever without source Ann Emergency Med 1994 24 836 41 10.1016/S0196-0644(94)70201-2 7978555

[B61] Latoszek-Berendsen A Talmon J de Clercq P Hasman A With good intentions Int J Med Inform 2007 76S S440 6 10.1016/j.ijmedinf.2007.05.012 17613271

[B62] Schünemann HJ Woodhead M Anzueto A Buist S MacNee W Rabe KF Hefner J A vision statement on guideline development for respiratory disease: the example of CPOD Lancet 2009 373 774 9 10.1016/S0140-6736(08)61347-1 18835641

[B63] Elwyn G O'Connor AM Bennett C Newcombe RG Politi M Durand MA Drake E Joseph-Williams N Khangura S Saarimaki A Sivell S Stiel M Bernstein SJ Col N Coulter A Eden K Härter M Rovner MH Moumjid N Stacey D Thomson R Whelan T Weijden T van der Edwards A Assessing the quality of decision support technologies using the International Patient Decision Aid Standards instrument (IPDASi) PLoS One 2009 4 3 e4705 Epub 2009 Mar 4 10.1371/journal.pone.0004705 19259269 PMC2649534

[B64] O'Connor AM Bennett C Stacey D Barry MJ Col NF Eden KB Do patient decision aids meet effectiveness criteria of the international patient decision aid standards collaboration? A systematic review and meta-analysis Med Dec Mak 2007 27 554 74 10.1177/0272989X07307319 17873255

[B65] Saltman DC Guidelines for every person J Eval Clin Pract 1998 4 1 9 10.1046/j.1365-2753.1998.t01-1-00001.x 9524908

